# Validation of an adapted Pediatric Sepsis Score in children admitted to PICU with invasive infection and sepsis: a retrospective analysis of a Dutch national cohort

**DOI:** 10.1186/s40560-022-00618-3

**Published:** 2022-06-07

**Authors:** Navin P. Boeddha, Luregn J. Schlapbach, Idse H. Visser, Nicolaas J. G. Jansen, SKIC (Dutch Collaborative PICU Research Network), Casper Bollen, Marc van Heerde, Douwe van der Heide, Richard Klein, Martin Kneyber, Jan-Willem Kuiper, Maaike Riedijk, Carin Verlaat, Dick van Waardenburg

**Affiliations:** 1 Intensive Care and Department of Pediatric Surgery, Erasmus MC-Sophia Children’s Hospital, University Medical Center Rotterdam, Rotterdam, The Netherlands; 2 Department of Pediatrics, Erasmus MC-Sophia Children’s Hospital, University Medical Center Rotterdam, Rotterdam, The Netherlands; 3 Child Health Research Centre, The University of Queensland, and Paediatric Intensive Care Unit, Queensland Children’s Hospital, Brisbane, Australia; 4 Pediatric and Neonatal Intensive Care Unit, University Children’s Hospital Zurich, Zurich, Switzerland; 5 Dutch Pediatric Intensive Care Evaluation, Medical Research Data Management, Deventer, The Netherlands; 6 Department of Pediatric Intensive Care, Wilhelmina Children’s Hospital, University Medical Center Utrecht, Utrecht, The Netherlands; 7 Department of Pediatrics, Beatrix Children’s Hospital, University Medical Center Groningen, Groningen, The Netherlands

**Keywords:** Child, Mortality, Organ dysfunction, Sepsis, Septic shock, Score

## Abstract

We validated an adapted form of the Pediatric Sepsis Score (aPSS), a disease-specific severity score available within 60 min of PICU admission, in children with invasive infection. aPSS consist of all components of PSS except lactate. aPSS predicted mortality in children with invasive infection (*n* = 4096; AUC 0.70 (95% CI 0.67–0.73)) and in children with sepsis (*n* = 1690; AUC 0.71 (0.67–0.76)). aPSS can be an adequate tool to predict outcome in children admitted to PICU with invasive infection or sepsis, especially in situations where lactate is not available within 60 min.

## To the editor,

The revised sepsis definitions in adults [1] highlight the need to identify patients with infection subject to substantially higher mortality. Early recognition of high-risk sepsis patients by organ dysfunction scores is key to select patients for specific therapies and for enrolment in trials.

While most organ dysfunction scores are based on the worst state within 24 h [2], the fulminant nature of pediatric sepsis warrants tools that can be applied to patients upon presentation. We previously developed the Pediatric Sepsis Score (PSS) [3], available within 60 min of PICU admission and predicting mortality superior to Paediatric Index of Mortality-2 (PIM2), also calculated within 60 min of PICU admission [4].

The PSS includes respiratory, cardiovascular, metabolic (lactate) and neurologic variables. As lactate is not always available within 60 min of PICU admission, we omitted this and studied an adapted form of PSS (aPSS).

This study aims to validate aPSS in an independent cohort and to compare its performance with PIM2.

## Materials and methods

A retrospective analysis of the Dutch Pediatric Intensive Care Evaluation (PICE) registry (www.pice.nl) which prospectively records all children admitted to the 8 Dutch PICUs. We included non-elective patients < 16 years, who were admitted to PICU from 2003 to 2016, when a diagnosis of any invasive infection or sepsis was registered in the principal and/or the first underlying diagnostic fields. Invasive infection includes meningitis, pneumonia/pneumonitis, peritonitis, necrotizing fasciitis, osteomyelitis, endocarditis, tracheitis, epiglottitis, sepsis, septic shock, or toxic shock. This coding system is similar to the ANZPIC registry diagnostic code list [5]. The aPSS was calculated as sum of scores allocated for each predictor; PaO2/FiO2 ratio (0 =  ≥ 300, 3 = 100–300, 5 =  < 100), ventilation during the first hour (0 = no, 3 = yes), systolic blood pressure (3 = age-specific hypotension), cardiac arrest (0 = no, 5 = yes), and pupils (0 = both reactive, 10 = both dilated, unresponsive) [3]. The primary outcome was PICU mortality. Patients were classified as “having an underlying condition” if a chronic condition was present in any diagnostic field including the associated diagnostic fields [6, 7]. The AUC of aPSS was compared with PIM2.

## Results

4096 children (57% male, median age 2 years (IQR 0–7y)) were admitted to PICU with any invasive infection, including a subgroup of 1690 patients with sepsis (56% male, median age 2 years (IQR 0-8y)). Of all children, 1987/4096 (49%) were mechanically ventilated in the first hour of admission, the median PICU length of stay was 3.9 days (IQR 1.6–8.5 days), with a mean predicted death rate of 6.7% (SD 11.7) as per PIM2 and an observed mortality of 8.0% (329/4096). In the subgroup of patients with sepsis, 794/1690 (47%) were mechanically ventilated in the first hour, the median PICU length of stay was 3.2 days (IQR 1.3–7.3d), with a mean predicted death rate of 8.9% (SD 14.5) as per PIM2 and an observed mortality of 12% (210/1690).

**Fig. 1:**
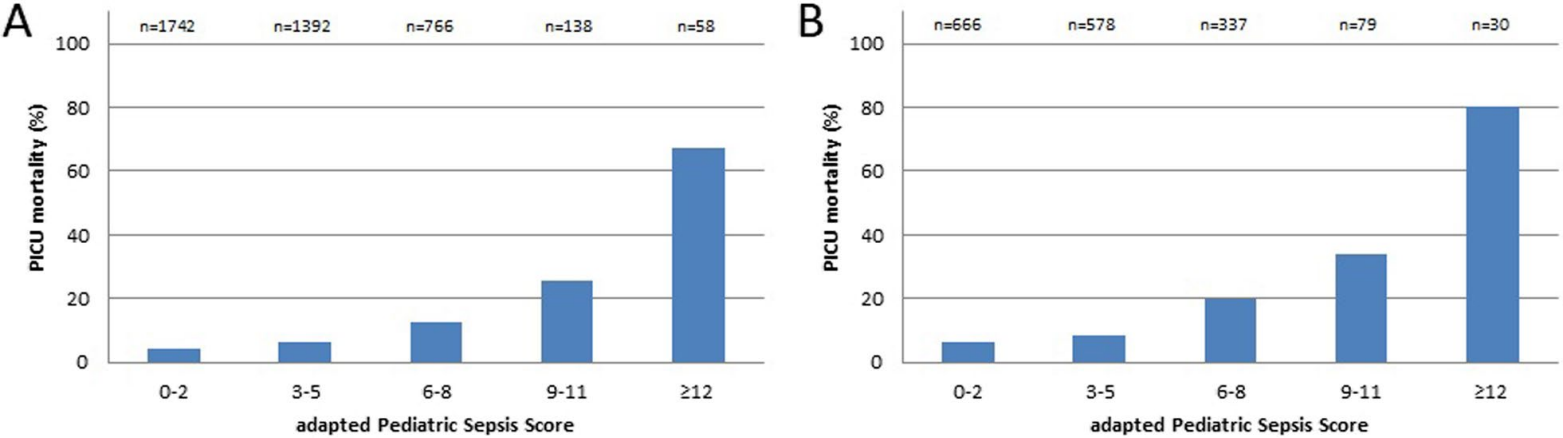
PICU mortality in children admitted to the Dutch PICUs is stratified by the adapted Pediatric Sepsis Score. **A** Children with any invasive infection (*n* = 4096. **B** Children with sepsis (*n* = 1690)

aPSS was correlated to mortality in children with any invasive infection (Spearman r = 0.20, p < 0.001) and in the subgroup of children with sepsis (Spearman *r* = 0.26, *p* < 0.001) (Fig. 1). This finding was present in both children without underlying conditions and with underlying conditions (*p* < 0.001).

In children with any invasive infection, aPSS predicted mortality with an AUC of 0.70 (95%-CI 0.67–0.73) (Table 1). In children with sepsis, aPSS predicted mortality with an AUC of 0.71 (95%-CI 0.67–0.76). aPSS discriminated better in children without underlying conditions than in children with underlying conditions. Comparing the aPSS with PIM2, the discrimination ability on the primary outcome was less in any invasive infection, but equal in sepsis.

**Table 1:** AUC for aPSS as compared with the PIM2 are shown for PICU mortality

Patient category	PICU mortality
Any invasive infection^a^ (*n* = 4096)	aPSS: 0.70 (0.67–0.73) PIM2: 0.74 (0.71–0.77)*
Any invasive infection^a^ without underlying conditions (*n* = 1922)	aPSS: 0.81 (0.77–0.86) PIM2: 0.85 (0.81–0.90)*
Any invasive infection^a^ with underlying conditions (*n* = 2174)	aPSS: 0.65 (0.61–0.69) PIM2: 0.68 (0.65–0.72)*
Sepsis^b^ (*n* = 1690)	aPSS: 0.71 (0.67–0.76) PIM2: 0.73 (0.69–0.77)
Sepsis^b^ without underlying conditions (*n* = 821)	aPSS: 0.83 (0.78–0.89) PIM2: 0.84 (0.79–0.90)
Sepsis^b^ with underlying conditions (*n* = 869)	aPSS: 0.65 (0.60–0.71) PIM2: 0.67 (0.61–0.72)

AUC with the respective 95%-confidence intervals are shown for the primary outcome (PICU mortality)

*p < 0.05 for comparison between aPSS and PIM2

^a^Invasive infection: meningitis, pneumonia/pneumonitis, peritonitis, necrotizing fasciitis, osteomyelitis, endocarditis, tracheitis, epiglottitis, sepsis, septic shock, or toxic shock as the principal PICU diagnosis or as the first underlying diagnosis

^b^Sepsis: sepsis, septic shock, or toxic shock as the principal PICU diagnosis or as the first underlying diagnosis

^a, b^ANZPIC registry diagnostic codes [5]

## Discussion

Improving treatment of children with suspected sepsis relies on accurate and rapid recognition of patients at higher risk of poor outcomes. Whereas PSS was developed in Australia and New Zealand, this independent validation demonstrates that aPSS performs adequately for mortality. aPSS seems an important addition as in many locations lactate is not available within 60 min of admission to a PICU.

Overall, the performance of aPSS in the Dutch validation dataset was lower compared to the performance of PSS reported in the Australian and New Zealand cohort, in which the PSS was derived. First, lactate was an independent predictor of mortality in the original dataset [3], and hence lack of lactate likely contributed to lower score performance. Second, different practices in coding strategies may affect patient severity. However, the findings demonstrate the importance of independent validation as the original score was developed within cohort.

Despite these limitations, this validation study demonstrates that aPSS could be a tool to detect organ dysfunction, to predict mortality, and can be used especially in situations where lactate is not available within 60 min. The discriminative performance of aPSS was less in invasive infections, but equally compared to PIM2 in sepsis.

Future studies should aim to validate the aPSS and full PSS including lactate more extensive.

## Data Availability

The datasets used and/or analysed during the current study are available from the corresponding author on reasonable request.

## Abbreviations

aPSS: Adapted Pediatric Sepsis Score
PICE: Pediatric intensive care evaluation
PIM2: Paediatric index of mortality-2
PSS: Pediatric Sepsis Score
SKIC: Dutch collaborative PICU research network
